# Utilization of immune checkpoint inhibitors for the treatment of cancer in Scotland, 2018–2024: A national retrospective cohort study

**DOI:** 10.1002/bcp.70700

**Published:** 2026-07-20

**Authors:** Tanja Mueller, Christine Crearie, Liya Lu, Jiafeng Pan, Fraser N. Sneden, David Martin, Amanj Kurdi, Catherine S. Thomson, Julie Clarke, Marion Bennie

**Affiliations:** ^1^ Strathclyde Institute of Pharmacy and Biomedical Sciences University of Strathclyde Glasgow UK; ^2^ Public Health Scotland Glasgow/Edinburgh UK; ^3^ NHS Greater Glasgow and Clyde Glasgow UK

**Keywords:** cancer, immune checkpoint inhibitors, Scotland, systemic anti‐cancer therapy, treatment uptake

## Abstract

**Aims:**

The aim of the study is to describe the use of immune checkpoint inhibitors (ICIs) for the treatment of cancer in Scotland.

**Methods:**

The retrospective observational cohort study included patients aged 18 years or older who commenced treatment with an ICI in Scotland between 1 January 2018 and 31 December 2024. The National Systemic Anti‐Cancer Therapy (SACT) dataset was used to identify patients and obtain details on ICI treatments and patient characteristics. Additional data sources providing information on comorbidities were linked deterministically via a unique patient identifier. All analyses were descriptive.

**Results:**

In total, 10 394 patients in Scotland initiated their first ICI treatment between 2018 and 2024; the number of patients initiating ICIs increased substantially year‐on‐year, from 764 patients in 2018 to 2233 patients in 2024. The most commonly prescribed ICI was pembrolizumab (56.8%, *n* = 5908), followed by nivolumab (16.4%, *n* = 1700) and atezolizumab (11.3%, *n* = 1170); treatment was most commonly initiated for lung and chest cancers (44.1%, *n* = 4585), skin cancer (18.8%, *n* = 1955) and urological cancers (14.8%, *n* = 1539). While the majority of patients (66.6%, *n* = 6923) initiated ICI monotherapy, combination therapies—most commonly ICI with chemotherapy (18.4%, n = 1910)—were also used. Overall, 6.6% of patients (*n* = 681) were on treatment for more than 2 years.

**Conclusions:**

With the continuous addition of new ICIs, alongside the ever‐expanding list of approved indications for their use, the observed increase in ICI use is expected to continue. This increasing use of ICIs may pose challenges to health services—for instance, an increase in immune‐related adverse effects—with potential implications for clinical practice and future research.

**What is known about this subject?**

- Immunotherapy represents a significant development in cancer treatment, potentially offering durable, long‐term remission.
- Immune checkpoint inhibitors such as nivolumab and pembrolizumab have been approved for a variety of cancer types.
- There is, however, limited evidence on how immune checkpoint inhibitors are being used generally in routine clinical practice.

**What this study adds?**

- The uptake of immune checkpoint inhibitors has been swift in Scotland.
- Findings are indicative of a growing diversification of immune checkpoint inhibitor use—including a noticeable increase in the share of patients treated with curable intent.
- Use of immune checkpoint inhibitors is expected to further increase substantially, with potential impact on health‐care resources.

## 1 INTRODUCTION

Cancer is one of the most common causes of death in many countries^1^ and is therefore high on the list of priorities of all stakeholders, not least those of patients, the health services, and governments. In 2023, an estimated 18.5 m patients were newly diagnosed with cancer globally, with approximately 10.4 m deaths because of cancer^2^; in Scotland alone—a country with a population of approximately 5.4 m people—the number of new cancer cases was just over 37 000 in 2023, with more than 16 000 deaths.^3^ Nevertheless, while cancer incidence is increasing globally,^4^ the cancer death rate has fallen in some countries over the last decades; in Scotland, for instance, from 328.5 per 100 000 population in 2015 to 288.9 per 100 000 in 2024.^3^ Today, half of patients diagnosed with cancer in the UK are expected to survive for at least 10 years, in contrast to just a quarter of patients in the 1970s.^5^

While better early detection of cancer as well as the targeting of modifiable risk factors (such as smoking) have certainly played a role in decreasing cancer mortality,^6^ improved survival of patients with many different cancer types, including melanoma and breast cancer, is also attributable to the availability of new treatment options such as targeted therapies and immunotherapies.^7^ Immunotherapies, in particular, represent a significant change in direction when it comes to cancer treatment. Unlike traditional chemotherapy, immunotherapy does not directly target cancer cells, but instead activates a patient's immune system which, in turn, attacks the cancer cells. Immune checkpoint inhibitors (ICIs), for example, interact with T cells, which are part of the body's immune system and responsible for the detection and disposal of infected or damaged cells (including cancer cells).^8^ T‐cell response is controlled by checkpoint proteins displayed on the T‐cell surface. The presence of large numbers of these checkpoint proteins prevents an effective immune response; by blocking these checkpoint proteins, ICIs therefore enable the body's immune system to recognize and eliminate cancer cells.^9^

The first ICI to gain market approval—first in the United States (US) and then across Europe—was ipilimumab, with initial approval for advanced (unresectable or metastatic) melanoma in 2011/2012; this was followed by nivolumab and pembrolizumab in 2014/2015, also for melanoma.^10^ Since then, several additional ICIs have been approved for a variety of indications, including lung cancer, renal cell carcinoma, bladder cancer, oesophageal cancer, breast cancer, colorectal cancer and Hodgkin lymphoma.^11^, ^12^ In addition, indications for already approved ICIs have been expanded; pembrolizumab, for instance, is now approved for a number of cancer types, from head and neck tumours to gynaecological cancers, including adjuvant and/or neo‐adjuvant treatments.^13^ For an overview of approved indications in Scotland, see Table S1.

The increasing availability of ICIs, for an ever‐increasing range of tumour types, has transformed the treatment landscape for advanced cancers, potentially offering durable, long‐term remission.^14^ While not necessarily always curable, cancer has become, for many patients, a chronic disease, with treatment given continuously over extended periods of time. Despite the undisputable positive impact of expanding the array of available cancer treatments on patients, this expansion is not without challenges. First and foremost, cancer treatment is becoming increasingly complex, with potential impacts on health services and clinical staff; modern cancer care requires substantial levels of staffing, and expanding role complexities for physicians, nurses, and pharmacists may exacerbate existing workforce pressures. Furthermore, the rising costs of new, innovative cancer therapies—such as ICIs—pose a significant challenge to the affordability of treatment.^15^ The increasing number of patients being treated for cancer, alongside the increasing average length of treatment duration, may therefore require the revision of existing cancer policies and/or treatment guidelines.

Nevertheless, although ICIs have been introduced more than a decade ago, there is limited evidence on how they are being used generally in routine clinical practice. Only a small number of publications offer insights into ICI uptake and utilization from a tumour‐agnostic point of view—for example, from Canada,^16^ Korea,^17^ and the United States^18^, ^19^—with the majority of studies thus far focussing on specific cancers, most commonly lung cancer^20^, ^21^ or urological cancers.^22^, ^23^ Consequently, the aim of this study was to describe the overall use of ICIs for the treatment of cancer in Scotland to provide further insights into the current treatment landscape and to inform future research.

## 2 METHODS

### 2.1 Setting

The National Health Service (NHS) Scotland is responsible for planning and delivering health care to the entire population, with a strong emphasis on patient‐centred, equitable and high‐quality health care funded primarily through taxation. Cancer treatment in Scotland is coordinated through three regional cancer networks: the West of Scotland Cancer Network (WoSCAN)^24^, the South East Scotland Cancer Network (SCAN)^25^ and the North Cancer Alliance (NCA).^26^ These three cancer networks work collaboratively to ensure consistent and equitable access to cancer services, from diagnosis and surgery to radiotherapy and systemic anti‐cancer therapies (SACT), including ICIs. SACT treatment guidelines are updated based on recommendations on new medicines/indications issued by the Scottish Medicines Consortium (SMC).^27^ Services are available free at the point of care for all residents in Scotland.

### 2.2 Study cohort

This study was designed as a retrospective observational cohort study covering the entirety of Scotland through collaboration of the three regional cancer networks. The study population included all adult patients aged ≥18 years in Scotland who received a first prescription of an ICI (avelumab, atezolizumab, cemiplimab, dostarlimab, durvalumab, ipilimumab, nivolumab, pembrolizumab), either alone or in combination with other SACT, for any tumour type between 1 January 2018 and 31 December 2024. Patients were included if they had not received any ICI previously, with a look‐back period to 2014; however, patients were included regardless of prior treatment with other SACT. The cohort inclusion period was selected based on the timing of medicine approval in Scotland and data availability, ensuring at least 6 months of follow‐up data after initial ICI prescription for all patients to provide insights into treatment duration. Patients were excluded if they were under 18 years of age, or if they commenced their first ICI treatment as part of a clinical trial.

### 2.3 Data sources

In Scotland, SACT is prescribed using specialist Chemotherapy Electronic Prescribing and Administration System (CEPAS) software, and captured in the National SACT Dataset for patients aged ≥16 years at the time of prescription.^28^ This National SACT dataset was used to identify patients included in this study. Details on the ICI treatment (e.g., drug name, treatment indication, appointment dates), as well as patient demographics, were also extracted from the National SACT dataset. Additional data sources included the Prescribing Information System (PIS)^29^ and the Scottish Morbidity Records general acute inpatients and day cases dataset (SMR01),^30^ providing information on comorbidities; death records from National Records of Scotland^31^ were used to censor treatment follow‐up. Data from these sources were linked deterministically using the unique patient identifier available in all health and care records in Scotland.

### 2.4 Definitions

#### 2.4.1 Index treatment, index date, treatment intent and duration of treatment

Index treatment was defined as the first ICI prescribed to a patient during the study period, as recorded in the National SACT dataset; in the dataset, treatment is prescribed by regimen, which includes ICI monotherapy and various combination treatments (e.g., ICI with chemotherapy, ICI with tyrosine kinase inhibitor). The index year (year ICI initiated) was the year of the index date that was defined as the date of first administration of the index ICI regimen.

ICI treatment may be prescribed for a specific disease indication (e.g., adjuvant cutaneous melanoma or metastatic breast cancer); or with a curable or non‐curable intent. Patients prescribed ICIs with a neo‐adjuvant/adjuvant disease indication or a curable treatment intent at the index date were defined as treated with curable intent, whereas patients prescribed ICIs with a metastatic disease indication or a non‐curable treatment intent at the index date were defined as treated with non‐curable intent.

Treatment duration was calculated as the time from the first to the last ICI prescription, plus the length of the final cycle; treatment duration was censored at patient death, the date of switching from the index ICI to a different SACT, or the study end date (30 June 2025), whichever occurred first. Patients were considered to be still on treatment if they had received a prescription for an ICI within 3 months prior to the study end date and were still alive.

#### 2.4.2 Patient characteristics

Patient age at the index date was calculated based on date of birth and appointment date; sex was a binary variable (male, female) as recorded on the prescribing record. The Scottish Index of Multiple Deprivation (SIMD) was used to define patient socio‐economic deprivation, categorized into quintiles from 1 (most deprived) to 5 (least deprived).^32^ Urban and rural status was classified using the eightfold Scottish Urban Rural Classification, based on settlement size and accessibility, ranging from 1 (large urban) to 8 (very remote rural).^33^ Eastern Cooperative Oncology Group Performance Status (ECOG PS)^34^ was used to assess patients' functional ability, ranging from 0 to 5, with lower scores indicating better functional status. Comorbidities were captured through two different methods: first, the number of medicines dispensed in community pharmacies (excluding prescriptions for vaccines, dressings and appliances) in the year preceding the index date; and second, by calculating a Charlson score based on hospital discharge records covering the 5‐year period prior to the index date (excluding the cancer of interest).^35^

### 2.5 Statistical analysis

All analyses were descriptive. Categorical variables (index ICI, index year, tumour group; patient sex, SIMD, urban/rural status, ECOG PS, number of medicines dispensed in community pharmacies [grouped as 0, 1–4, 5–9, 10 or more], Charlson score [grouped as 0, 1, 2 or higher]) were summarized using frequencies and percentages. Similarly, treatment duration was summarized using mutually exclusive categories (<6 months, 6–12 months, 12–24 months, >24 months). To illustrate ICI prescribing trends over time, treatments were further stratified by index year, ICI drug, and tumour group.

## 3 RESULTS

### 3.1 Patient baseline characteristics

Overall, 10 394 patients with cancer in Scotland who met the inclusion criteria initiated their first ICI treatment between 1 January 2018 and 31 December 2024 (Figure 1).

**FIGURE 1:**
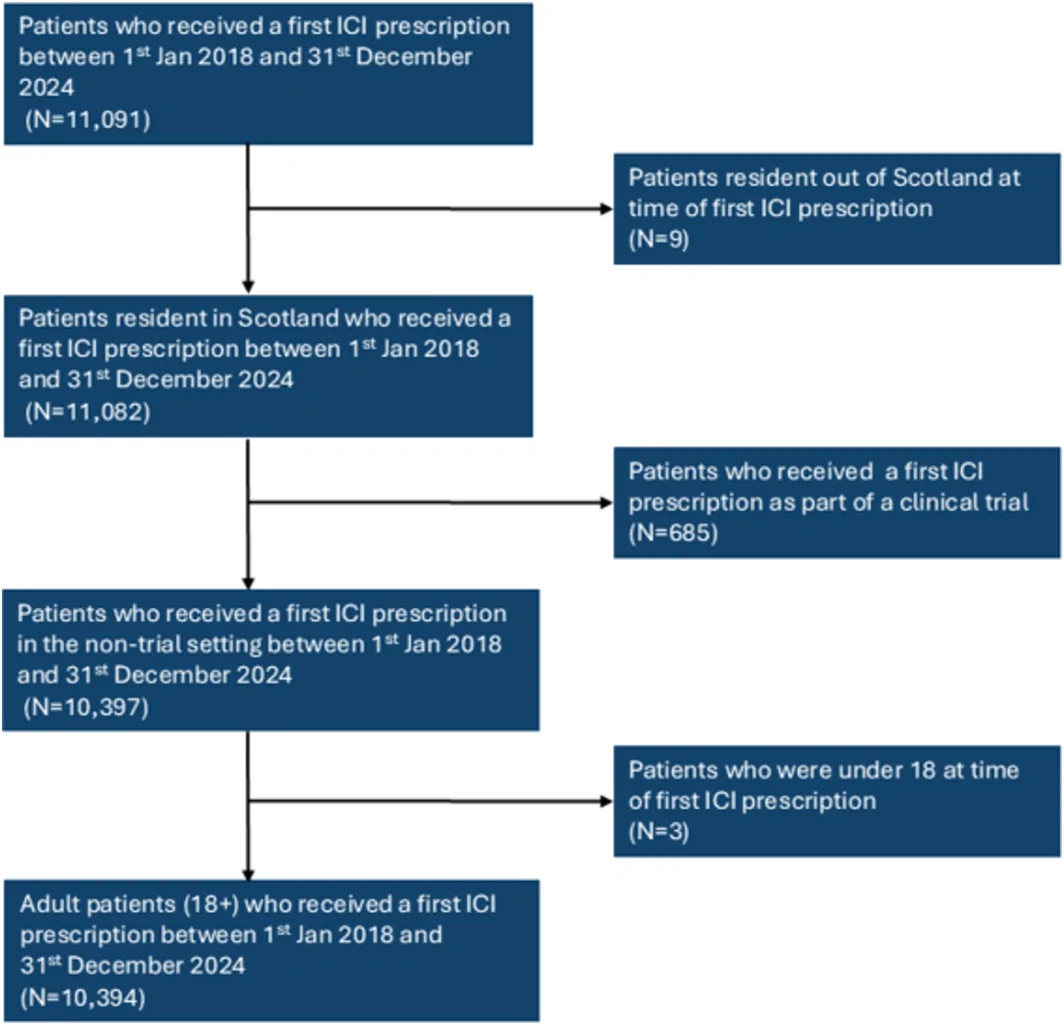
Cohort identification – patients (18 years of age or older) initiating an immune checkpoint inhibitor during the study period (January 2018 to December 2024).

The median age at ICI initiation was 66 years (IQR 58–73), and a little over half the patients (*n* = 5617, 54.0%) were male; the majority of patients lived in urban areas (*n* = 7283, 70.1%), received >5 medicines from community pharmacies (n = 8544, 82.2%) and had a Charlson score of 0 (*n* = 6483, 62.4%). At the time of ICI initiation, patients predominantly had low ECOG PS scores of 0 and 1 (*n* = 8984, 86.4%); a third of the patients (*n* = 3494, 33.6%) had received SACT prior to initiating treatment with an ICI. For details, see Table 1.

**TABLE 1:** Patient baseline characteristics (*n* = 10 394).

Patient characteristic	Number of patients (%)
**Sex**	
Male	5617 (54.0)
Female	4777 (46.0)
**Age [years]**	
Median (IQR)	66 (58–73)
Range	18–96
**SIMD**	
1 (most deprived)	2255 (21.7)
2	2155 (20.7)
3	2157 (20.8)
4	1952 (18.8)
5 (least deprived)	1844 (17.7)
Unknown	31 (0.3)
**Urban/rural status**	
1 (Large urban area)	4010 (38.6)
2 (Other urban area)	3273 (31.5)
3 (Accessible small town)	1016 (9.8)
4 (Remote small town)	76 (0.7)
5 (Very remote small town)	182 (1.8)
6 (Accessible rural area)	1274 (12.3)
7 (Remote rural area)	217 (2.1)
8 (Very remote rural area)	315 (3.0)
Unknown	31 (0.3)
**ECOG PS**	
0	3492 (33.6)
1	5492 (52.8)
2 or higher	598 (5.8)
Unknown	812 (7.8)
**SACT prior to ICI initiation**	
Yes	3494 (33.6)
No	6900 (66.4)
**Number of medicines dispensed in community pharmacy**	
0	248 (2.4)
1–4	1602 (15.4)
5–9	3090 (29.7)
10+	5454 (52.5)
**Charlson score**	
0	6483 (62.4)
1	1709 (16.4)
2+	2185 (21.0)
No hospital record	17 (0.2)

Abbreviations: ECOG PS, Eastern Cooperative Oncology Group Performance Status; ICI, immune checkpoint inhibitor; IQR, interquartile range; SACT, systemic anti‐cancer therapy; SIMD, Scottish Index of Multiple Deprivation.

### 3.2 Index treatments

Patients initiated ICIs across a variety of tumour groups, most frequently lung and chest (*n* = 4585, 44.1%), skin (*n* = 1955, 18.8%) and urology (*n* = 1539, 14.8%). Pembrolizumab (*n* = 5908, 56.8%) was the most commonly prescribed ICI, followed by nivolumab (*n* = 1700, 16.4%) and atezolizumab (*n* = 1170, 11.3%). ICIs were most frequently administrated as a monotherapy (*n* = 6923, 66.6%), followed by ICI combined with chemotherapy (*n* = 1910, 18.4%; see Table 2).

**TABLE 2:** Index drug and indication for treatment (*n* = 10 394).

Treatment characteristic	Number of patients (%)
**Index year**	
2018	764 (7.4)
2019	1033 (9.9)
2020	1243 (12.0)
2021	1501 (14.4)
2022	1640 (15.8)
2023	1980 (19.0)
2024	2233 (21.5)
**Index ICI**	
Pembrolizumab	5908 (56.8)
Nivolumab	1700 (16.4)
Atezolizumab	1170 (11.3)
Ipilimumab + nivolumab	782 (7.5)
Durvalumab	402 (3.9)
Cemiplimab	214 (2.1)
Avelumab	137 (1.3)
Dostarlimab	52 (0.5)
Nivolumab + relatlimab	24 (0.2)
Durvalumab + tremelimumab	3 (<0.1)
Ipilimumab	2 (<0.1)
**ICI regimen type**	
ICI monotherapy	6923 (66.6)
ICI + chemotherapy	1910 (18.4)
ICI combination^a^	809 (7.8)
ICI + TKI	602 (5.8)
ICI + VEGF^b^	150 (1.4)
**Tumour group (indication for prescribing)**	
Lung and chest	4585 (44.1)
Skin	1955 (18.8)
Urology	1539 (14.8)
Upper GI	671 (6.5)
Head and neck	625 (6.0)
Gynaecology	330 (3.2)
Breast	324 (3.1)
Lower GI	302 (2.9)
Haematology	45 (0.4)
Other^c^	18 (0.2)
**Treatment intent**	
Curable intent	1688 (16.2)
Non‐curable intent	8706 (83.8)

Abbreviations: GI, gastrointestinal; ICI, immune checkpoint inhibitor; TKI, tyrosine kinase inhibitor; VEGF, vascular endothelial growth factor.

^a^ Patients treated with more than one immune checkpoint inhibitor simultaneously (e.g., ipilimumab with nivolumab).

^b^ With or without chemotherapy.

^c^ Comprises endocrine tumours, sarcoma, tumours of the Central Nervous System, and cancers of unknown primary.

### 3.3 ICI prescribing trends over time

During the study period, the number of patients initiating their first ICI increased substantially, from 764 patients in 2018 to 2233 in 2024—a relative increase of 192.3%, with an average annual change of +19.8% (Figure 2). Pembrolizumab was consistently the most prescribed ICI, with a steady increase from 410 patients in 2018 to 1239 in 2024; increases in the number of patients treated with pembrolizumab per year, in comparison to the preceding year, were most apparent in 2020 (from 455 patients in 2019 to 783 patients in 2020), 2021 (783 to 896) and 2023 (928 to 1197), coinciding with SMC recommendations expanding approved indications in melanoma, lung cancer, and breast cancer. Patient numbers for other ICIs showed either more modest increases (atezolizumab, ipilimumab + nivolumab, durvalumab, cemiplimab, avelumab) or fluctuating use over time (nivolumab). The ICI combination therapies of nivolumab + relatlimab and durvalumab + tremelimumab were used for the first time in 2024; ipilimumab monotherapy use was very rare. Among the different regimen types, the combination of ICI with chemotherapy showed the largest relative change during the study period, from two patients in 2018 to 561 patients in 2024. Table S2 provides further details of use by drug over time.

**FIGURE 2:**
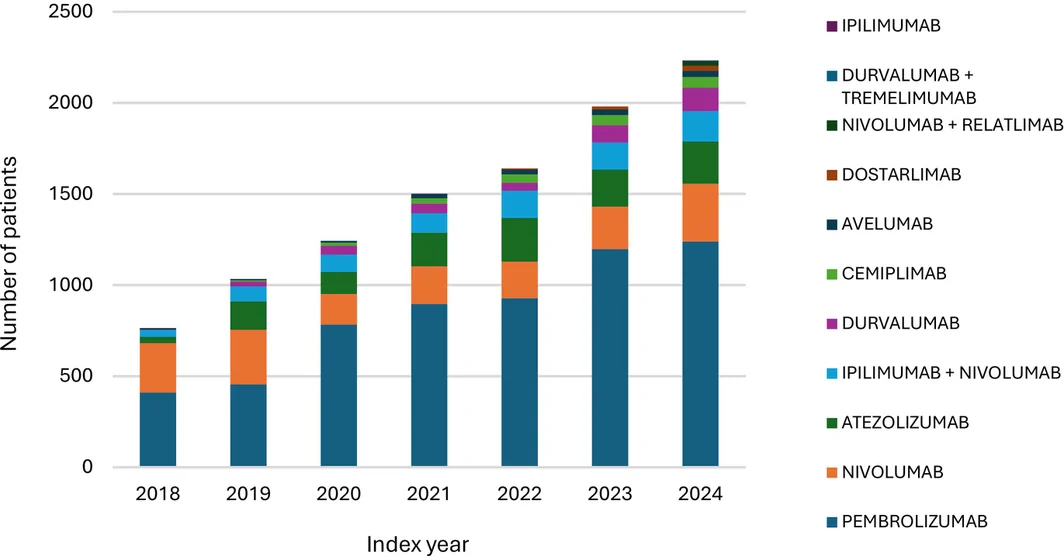
Uptake of immune checkpoint inhibitors in Scotland over time by drug, 2018–2024.

While the most common cancer among patients was lung cancer in all years, overall accounting for 44.1% of the total cohort (*n* = 4585), the expansion of licensed indications resulted in an increase of patients being treated for various other cancers during the study period. Increases in ICI use were most prominent for upper gastrointestinal tract cancer and breast cancer, coinciding with several SMC recommendations in these disease areas issued between 2022 and 2024 (Figure 3). The patients treated with curable intent increased from 3.4% in 2018 to 23.8% in 2024. For additional details on ICI use by tumour group and treatment intent over time, see Table S3.

**FIGURE 3:**
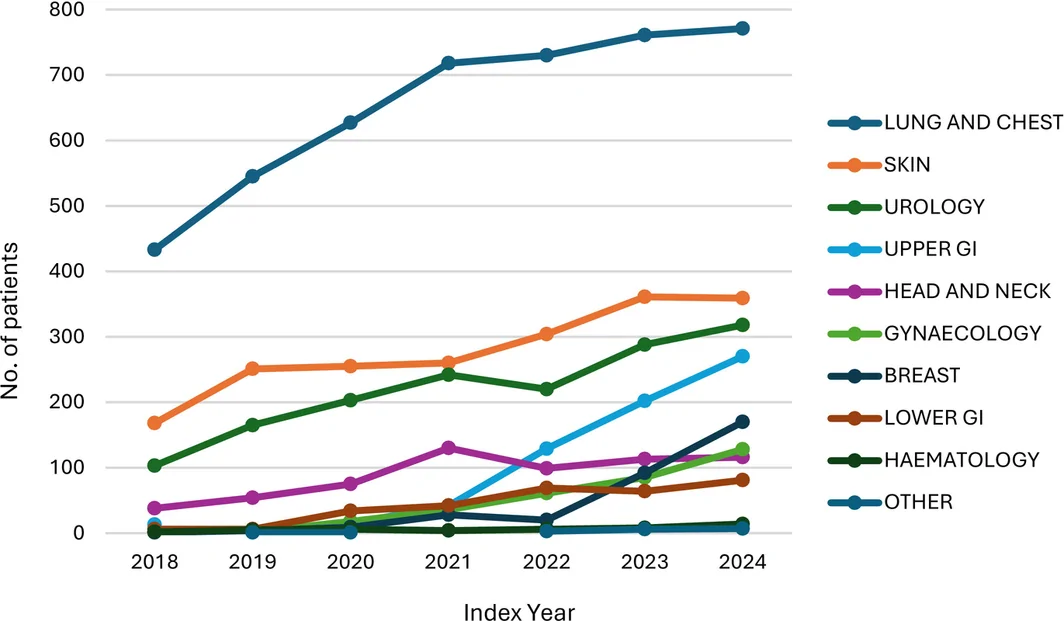
Uptake of immune checkpoint inhibitors over time in Scotland by tumour type, 2018–2024.

### 3.4 Treatment duration

Just over half the patients (*n* = 5645, 54.3%) were on treatment for less than 6 months, while treatment duration exceeded 2 years in 6.6% of patients in the full cohort (*n* = 681). However, treatment duration differed by treatment intent (curable *vs*. non‐curable) as well as by drug. Overall, both very short (<6 months) and very long (> 24 months) treatment durations were more common among patients with metastatic disease; see Table 3 for details.

**TABLE 3:** Treatment duration by index drug and treatment intent at initiation (*n* = 10 394).

Number of patients (%)	< 6 months	6–12 months	12 months – 24 months	> 24 months	Total
***Study cohort***					
Overall	5645 (54.3)	2367 (22.8)	1701 (16.4)	681 (6.6)	10 394
***Curable intent (adjuvant/neo‐adjuvant)***					
Pembrolizumab	350 (36.4)	250 (26.0)	354 (36.8)	7 (0.7)	962
Nivolumab	141 (40.5)	153 (44.0)	51 (14.7)	3 (0.9)	348
Durvalumab	104 (39.5)	109 (41.4)	50 (19.0)	‐	263
Atezolizumab	22 (41.5)	23 (43.4)	8 (15.1)	‐	53
Ipilimumab + nivolumab	25 (58.1)	6 (13.6)	5 (11.4)	7 (15.9)	44
Dostarlimab	4 (44.4)	5 (55.6)	‐	‐	9
Cemiplimab	3 (50.0)	3 (50.0)	‐	‐	6
Avelumab	3 (60.0)	‐	‐	2 (40.0)	5
Overall	652 (38.6)	549 (32.5)	468 (27.7)	19 (1.1)	1688
***Non‐curable intent (metastatic)***					
Pembrolizumab	2695 (54.5)	1036 (20.9)	771 (15.6)	445 (9.0)	4947
Nivolumab	875 (64.7)	249 (18.4)	159 (11.8)	69 (5.1)	1352
Atezolizumab	706 (63.2)	258 (23.1)	106 (9.5)	47 (4.2)	1117
Ipilimumab + nivolumab	444 (60.1)	129 (17.4)	101 (13.6)	65 (8.8)	739
Cemiplimab	98 (47.1)	44 (21.2)	48 (23.1)	18 (8.7)	208
Durvalumab	71 (51.1)	50 (36.0)	17 (12.2)	1 (0.7)	139
Avelumab	71 (53.8)	25 (18.9)	21 (15.9)	15 (11.4)	132
Dostarlimab	17 (39.5)	14 (32.6)	10 (23.3)	2 (4.7)	43
Nivolumab + relatlimab	12 (50.0)	12 (50.0)	‐	‐	24
Durvalumab + tremelimumab	2 (66.6)	1 (33.3)	‐	‐	3
Ipilimumab	2 (100.0)				2
Overall	4993 (57.4)	1818 (20.9)	1233 (14.2)	662 (7.6)	8706

## 4 DISCUSSION

The development of ICIs has introduced new, promising opportunities to treat patients with a variety of different cancers, and their uptake has accordingly been swift. In Scotland, the number of patients starting treatment with ICIs for the first time is continually rising and has reached 2233 in 2024, almost trebling over the study period. Among the available ICIs, pembrolizumab is currently the most widely used, accounting for approximately 57% of the ICI treatment initiations over the study period; patients with lung cancer represent the single largest group of ICI‐treated patients, representing 44% of the study cohort.

While a range of cohort studies have been published on the use of ICIs in specific tumour groups, for example, bladder cancer,^22^ renal cell cancer,^23^ mesothelioma^36^ or lung cancer,^20^, ^21^, ^37^ observational studies assessing the uptake and utilization of ICIs for unselected cohorts of patients with cancer are relatively scarce—despite these being helpful additions to the evidence base by providing a general overview of current clinical practice, thereby highlighting future areas of interest as well as potentially informing service planning. A study conducted in Canada using administrative health records to summarize ICI uptake between 2011 and 2019 provided results in line with those obtained in this study: ICI use has increased substantially over time, the most used ICI was pembrolizumab, and patients with lung cancer constituted the largest group within the cohort of patients treated with ICIs.^16^ Similarly, a US study analysing data from the National Cancer Database up to 2020 observed a substantial increase in the use of ICIs across various tumour groups over time^19^; in an earlier study using the US data from 2011 to 2016, lung cancer was the most common indication for ICI treatment, although nivolumab was the most commonly prescribed ICI.^18^ National health insurance data from Korea, covering the period 2017–2022, also indicated a steady increase in ICI use, with an expanding range of indications; while lung cancer was the most common indication, the study highlighted, in particular, the sharp increase in ICI use for liver/biliary tract cancer, as well as a substantial increase in the use of combination therapy (either concurrent use of more than one ICI, or with other SACT).^17^ Taken together, these studies confirm that ICIs are occupying an increasingly relevant position in cancer treatment, at least in high‐income countries. Furthermore, findings are clearly indicative of a growing diversification of ICI use—albeit with some regional differences in the tumour groups treated and the individual ICIs used.

The comparatively large proportion of patients with melanoma among all ICI‐treated patients in Scotland is potentially because of the early approval of ICIs for this indication, alongside the previously limited treatment options for advanced melanoma prior to the approval of ICIs; this is in line with findings from a Canadian study, where the share of patients being treated with ICIs among all those diagnosed with this cancer was highest in melanoma.^16^ In contrast, patients with lung cancer representing the largest patient group among ICI‐treated patients across studies are likely attributable to lung cancer being one of the most common cancers globally.^38^ With lung cancer incidence continuing to be high, and still predicted to rise in many countries in the decades to come,^38^ ICI use is expected to further increase substantially, especially keeping in mind the increasing familiarity of clinicians with using ICIs and the relative lack of other highly effective treatments for lung cancer^39^; this may raise questions related to, for example, equal access to treatment (considering financial challenges in many settings) or workforce planning and staff training.^40^

Changes in treatment guidelines, triggered by changes in approved indications for individual ICIs, are a possible explanation for the observed diversification of ICI use, especially in the latter years of the study period. For instance, pembrolizumab has been approved for neoadjuvant/adjuvant use in patients with breast cancer in Scotland in 2023,^41^ coinciding with a significant increase in observed use in 2023 compared to previous years. In contrast to this present study, earlier tumour‐agnostic studies only included patients with non‐curable intent, that is, with metastatic disease,^18^ stage 4 cancer^16^, ^19^ or where treatment intent was palliative^16^—in line with treatment eligibility at the time of study conduct. As initial approvals for ICIs were for advanced disease, patients with curable treatment intent (adjuvant and/or neo‐adjuvant treatment) would not have been present in early studies. Although patients with curable intent comprised only 16.2% of the cohort overall (most of which being patients with skin cancer), the share of patients treated with curable intent has already increased substantially during the study period (from 3.4% to 23.8%) and is expected to increase further in line with expanding approved indications for use.

Alongside the diversification of indications and the increasing share of patients being treated with curative intent, duration of treatment is a topic of interest in the context of ICI treatment. While the optimal treatment duration of ICIs is still subject to discussion,^42^ adjuvant treatment with pembrolizumab, for instance, is generally given for 1 year; patients treated with non‐curable intent undergo regular scans and may switch treatment earlier (e.g., after 3 or 6 months) if the cancer is progressing. Alternatively, treatment may be stopped early because of toxicities—with immune‐related adverse events being a particular challenge.^43^ Although follow‐up data were only available until June 2025 and was therefore limited to 6 months for some patients, study findings indicate that many patients stop treatment early (i.e., after less than 6 months); unfortunately, data on disease progression was not available for this study, and it therefore remains unknown why patients might have stopped treatment early. Interestingly, although 28.9% of patients treated with curable intent and 21.8% of patients treated with non‐curable intent were on treatment for longer than 1 year, overall, 8.7% of patients were on treatment for more than 2 years (1.1% and 7.6%, respectively) despite 2‐year stopping rules being in place for some ICIs in Scotland (e.g., nivolumab and pembrolizumab in some indications). Consequently, further research investigating the duration of treatment and reasons for stopping (or switching), as well as the impact of fixed or continuous duration of treatment on patient outcomes, is warranted.

### 4.1 Strengths and limitations

The main strength of this study is the coverage and quality of the data used for analysis. Data were available for all adult patients treated in Scotland during the study period, regardless of tumour type, treatment regimen or any patient‐related characteristics; in addition, as national‐level data in Scotland are collected in routine care for clinical (e.g., National SACT dataset), administrative (e.g., death records) or payment purposes (e.g., prescriptions dispensed in primary care), data completeness is high. Data quality assurance procedures are in place to ensure high quality of routinely collected data. Nevertheless, certain limitations need to be kept in mind. First and foremost, ICIs are only one treatment option among several (including other SACT as well as surgery and radiotherapy), and comprehensive information on other treatment options was not available for this study; hence, findings do not extend to patients who did not receive an ICI as part of their treatment regimen. With no denominator of all patients being treated for any cancers, it remains uncertain whether the increasing use of ICIs is because of increasing cancer incidence or solely attributable to changes in treatment preferences. Furthermore, because of censoring, treatment duration may have been underestimated, and reasons for stopping ICI treatment were unknown (e.g., disease progression or side effects). Finally, assessing the effectiveness and/or safety of different treatment options was outside the scope of this project.

## 5 CONCLUSION

With the continuous addition of new ICIs, alongside the ever‐expanding list of approved indications for their use, the increased use of ICIs for the treatment of a wide range of cancers is expected to continue. While the availability of ICIs is beneficial for patients, their widespread implementation into clinical practice may come with new challenges, including potential impacts on staff training and workload.

## AUTHOR CONTRIBUTIONS

All authors contributed to the conception and design of the work. Tanja Mueller and Christine Crearie drafted the initial manuscript, with input from all authors. All authors critically reviewed and revised the draft, and approved the final manuscript.

## CONFLICT OF INTEREST STATEMENT

The authors have no conflicts of interest to declare.

## Supporting information


**Table S1:** Indication and stopping rules for immune checkpoint inhibitors in Scotland (www.scottishmedicines.org.uk).
Table S2: Utilization of immune checkpoint inhibitors over time, by index drug (N = 10 394).
Table S3: Utilization of immune checkpoint inhibitors over time, by cancer type and treatment intent (N = 10 394).

## Data Availability

The data that support the findings of this study are available from the data controller, subject to approval (https://www.researchdata.scot/). Some data may not be publicly available because of ethical restrictions.
