# Supplementary material for: Utilization of immune checkpoint inhibitors for the treatment of cancer in Scotland, 2018–2024: A national retrospective cohort study
Source: Br J Clin Pharmacol. 2026 Jul 20;92(11):3972–81. doi: 10.1002/bcp.70700 (PMC13714622; doi:10.1002/bcp.70700)
Supplement: Supplementary file 1 [file BCP-92-3972-s001.docx]

**Supplementary material**

Table S1: Indication and stopping rules for immune checkpoint inhibitors in Scotland (www.scottishmedicines.org.uk)

| **Medicine** | **Indication (month/year of SMC decision)** | **Stopping rule** |
| --- | --- | --- |
| atezolizumab (Tecentriq) | Monotherapy: treatment for locally advanced or metastatic *NSCLC* after prior chemotherapy; patients with EGFR activating mutations or ALK-positive tumour mutations should also have received targeted therapy before receiving atezolizumab (06.2018) | Subject to a 2-year clinical stopping rule |
|  | Monotherapy: first-line treatment of metastatic *NSCLC* where tumours have a PD-L1 expression ≥50% tumour cells or ≥10% tumour-infiltrating immune cells and do not have EGFR mutant or ALK-positive NSCLC (10.2021) | Until disease progression or unmanageable toxicity |
|  | Monotherapy: adjuvant treatment following complete resection for adult patients with Stage II to IIIA *NSCLC* where tumours have PD-L1 expression on ≥50% of tumour cells and whose disease has not progressed following platinum-based adjuvant chemotherapy (07.2022) | Treatment is continued for one year unless there is disease recurrence or unacceptable toxicity |
|  | Combination: with carboplatin and etoposide for first-line treatment of adults with *ES-SCLC* (10.2020) | Until disease progression or unmanageable toxicity |
|  | Combination: with nab-paclitaxel for treatment of unresectable locally advanced or metastatic *TNBC* where tumours have PD-L1 expression ≥1% and with no prior chemotherapy for metastatic disease (10.2020) | Until disease progression or unmanageable toxicity |
|  | Combination: with bevacizumab for the treatment of adult patients with advanced or unresectable *HCC* with no prior systemic therapy (06.2021) | Until loss of clinical benefit or unmanageable toxicity |
| avelumab (Bavencio) | Monotherapy: treatment of adult patients with *mMCC* (04.2018) | Until disease progression or unacceptable toxicity |
|  | Monotherapy: first-line maintenance treatment of adult patients with locally advanced or metastatic *urothelial carcinoma* who are progression-free following platinum-based chemotherapy (07.2021) | Until disease progression or unacceptable toxicity |
|  | Combination: with axitinib for first-line treatment of adult patients with advanced *RCC* (09.2020) | Until disease progression or unacceptable toxicity |
| cemiplimab (Libtayo) | Monotherapy: treatment of adult patients with metastatic or locally advanced *CSCC* who are not candidates for curative surgery or curative radiation (11.2023) | Until disease progression or unacceptable toxicity |
|  | Monotherapy: treatment of adult patients with recurrent or metastatic *cervical cancer* with disease progression on or after platinum-based chemotherapy (10.2025) | Until disease progression or unacceptable toxicity |
| dostarlimab (Jemperli) | Monotherapy: treatment of adult patients with dMMR/MSI-H recurrent or advanced *EC* that has progressed on or following prior treatment with a platinum-containing regimen (02.2022) | Until disease progression or unacceptable toxicity |
|  | Combination: with platinum-containing chemotherapy for treatment of adult patients with dMMR/MSI-H primary advanced or recurrent *EC* and who are candidates for systemic therapy (03.2024) | Until disease progression, unacceptable toxicity or for a duration of up to 3 years |
| durvalumab (Imfinzi) | Monotherapy: treatment of locally advanced, unresectable *NSCLC* in adults whose tumours express PD-L1 on ≥1% of tumour cells and whose disease has not progressed following platinum-based chemoradiation therapy (10.2019) | Until disease progression or unacceptable toxicity, or a maximum of 12 months |
|  | Combination: with gemcitabine and cisplatin for the first-line treatment of adults with locally advanced, unresectable, or metastatic *biliary tract cancer* (10.2023) | Until disease progression or unacceptable toxicity |
|  | Combination: with etoposide and either carboplatin or cisplatin for the first-line treatment of adults with *ES-SCLC* (01.2025) | Until disease progression or unacceptable toxicity |
|  | Combination: with carboplatin and paclitaxel for first-line treatment of adults with primary advanced or recurrent *EC* who are candidates for systemic therapy, followed by maintenance treatment with: 1) durvalumab as monotherapy in EC that is dMMR; 2) durvalumab in combination with olaparib in EC that is pMMR (09.2025) | Until disease progression or unacceptable toxicity |
|  | Combination: with tremelimumab for first-line treatment of adults with advanced or unresectable *HCC* (10.2025) | Until disease progression or unacceptable toxicity |
| ipilimumab (Yervoy) | Monotherapy: treatment of advanced (unresectable or metastatic) *melanoma* in adults who have received prior therapy (03.2013) | For a total of four doses |
|  | Monotherapy: first-line treatment of advanced (unresectable or metastatic) *melanoma* in adults (10.2014) | For a total of four doses |
|  | Monotherapy: treatment of advanced (unresectable or metastatic) *melanoma* in adolescents 12 years of age and older (09.2018) | - |
| nivolumab (Opdivo) | Monotherapy: treatment of locally advanced or metastatic squamous *NSCLC* after prior chemotherapy in adults (06.2016) | As long as clinical benefit is observed or until treatment is no longer tolerated |
|  | Monotherapy: treatment of advanced (unresectable or metastatic) *melanoma* in adults previously untreated with ipilimumab (07.2016) | As long as clinical benefit is observed or until treatment is no longer tolerated |
|  | Monotherapy: treatment of locally advanced or metastatic non-squamous *NSCLC* after prior chemotherapy in adults (09.2016) | Subject to a two-year clinical stopping rule |
|  | Monotherapy: treatment of advanced *RCC* after prior therapy in adults (05.2017) | As long as clinical benefit is observed or until treatment is no longer tolerated |
|  | Monotherapy: treatment of adult patients with relapsed or refractory *cHL* after ASCT and treatment with brentuximab vedotin (06.2017) | As long as clinical benefit is observed or until treatment is no longer tolerated |
|  | Monotherapy: treatment of *SCCHN* in adults progressing on or after platinum-based therapy (08.2017) | Subject to a two-year clinical stopping rule |
|  | Monotherapy: adjuvant treatment of adults with *melanoma* with involvement of lymph nodes or metastatic disease who have undergone complete resection (11.2018) | For a maximum duration of 12 months |
|  | Monotherapy: treatment of adult patients with unresectable advanced, recurrent or metastatic *OSCC* after prior fluoropyrimidine- and platinum-based combination chemotherapy (07.2021) | As long as clinical benefit is observed or until treatment is no longer tolerated |
|  | Monotherapy: adjuvant treatment of adult patients with completely resected *oesophageal or GOJ cancer* who have residual pathologic disease following prior neoadjuvant chemoradiotherapy (04.2022) | Maximum treatment duration for adjuvant therapy is 12 months |
|  | Monotherapy: adjuvant treatment of adults with *MIUC* with tumour cell PD-L1 expression ≥1%, who are at high risk of recurrence after undergoing radical resection of MIUC (01.2023) | Maximum treatment duration for adjuvant therapy is 12 months |
|  | Combination: with ipilimumab for first-line treatment of advanced (unresectable or metastatic) *melanoma* in adults (10.2016) | As long as clinical benefit is observed or until treatment is no longer tolerated |
|  | Combination: with ipilimumab for first-line treatment of adult patients with intermediate/poor-risk advanced *RCC* (05.2019) | As long as clinical benefit is observed or until treatment is no longer tolerated |
|  | Combination: with ipilimumab for treatment of adult patients with dMMR or MSI-H metastatic *CRC* after prior fluoropyrimidine-based combination chemotherapy (11.2021) | As long as clinical benefit is observed or until treatment is no longer tolerated |
|  | Combination: with ipilimumab for first-line treatment of adult patients with unresectable malignant pleural *mesothelioma* (01.2022) | For up to 24 months in patients without disease progression |
|  | Combination: with fluoropyrimidine- and platinum-based combination chemotherapy for first-line treatment of adult patients HER2-negative advanced or metastatic *gastric, GOJ or oesophageal adenocarcinoma* whose tumours express PD-L1 with a CPS ≥5 (08.2022) | Until disease progression, unacceptable toxicity, or up to 24 months in patients without disease progression |
|  | Combination: with fluoropyrimidine- and platinum-based combination chemotherapy for first-line treatment of adult patients with unresectable advanced, recurrent or metastatic *OSCC* with tumour cell PD-L1 expression ≥1% (05.2023) | Until disease progression, unacceptable toxicity, or up to 24 months in patients without disease progression |
|  | Combination: with platinum-based chemotherapy for the neoadjuvant treatment of resectable (tumours ≥4 cm or node positive) *NSCLC* in adults (12.2023) | For three cycles |
|  | Combination: with ipilimumab for first-line treatment of adult patients with unresectable or metastatic dMMR or MSI-H metastatic *CRC* (12.2025) | Until disease progression, unacceptable toxicity or up to 24 months in patients without disease progression |
| Nivolumab / relatlimab (Opduolag) | Combination: first-line treatment of advanced (unresectable or metastatic) *melanoma* in adults and adolescents 12 years of age and older (06.2024) | As long as clinical benefit is observed or until treatment is no longer tolerated |
| Pembrolizumab (Keytruda) | Monotherapy: treatment of advanced (unresectable or metastatic) *melanoma* in adults previously untreated with ipilimumab (10.2015) | Until disease progression or unacceptable toxicity |
|  | Monotherapy: treatment of locally advanced or metastatic *NSCLC* in adults whose tumours express PD-L1 and who have received at least one prior chemotherapy regimen (12.2016) | Subject to a two-year clinical stopping rule |
|  | Monotherapy: first-line treatment of metastatic *NSCLC* in adults whose tumours express PD-L1 with a ≥50% TPS with no EGFR or ALK-positive tumour mutations (06.2017) | Subject to a two-year clinical stopping rule |
|  | Monotherapy: treatment of locally advanced or metastatic *urothelial carcinoma* in adults who have received prior platinum-containing chemotherapy (01.2018) | Subject to a two-year clinical stopping rule |
|  | Monotherapy: adult patients with relapsed or refractory *cHL* who have failed ASCT and brentuximab vedotin, or who are transplant-ineligible and have failed brentuximab vedotin (02.2018) | Subject to a two-year clinical stopping rule |
|  | Monotherapy: adjuvant treatment of adults with Stage III *melanoma* and lymph node involvement who have undergone complete resection (04.2019) | Until disease recurrence, unacceptable toxicity, or for a duration of up to one year |
|  | Monotherapy: first-line treatment of metastatic or unresectable recurrent *SCCHN* in adults whose tumours express PD-L1 with a CPS ≥1 (08.2020) | Subject to a two-year clinical stopping rule |
|  | Monotherapy: first-line treatment of MSI-H or dMMR *CRC* in adults (08.2021) | Subject to a two-year clinical stopping rule |
|  | Monotherapy: treatment of adult and paediatric patients aged 3 years and older with relapsed or refractory *cHL* who have failed ASCT or following at least two prior therapies when ASCT is not a treatment option (10.2021) | Subject to a two-year clinical stopping rule |
|  | Monotherapy: adjuvant treatment of adults with *RCC* at increased risk of recurrence following nephrectomy, or following nephrectomy and resection of metastatic lesions (09.2022) | Until disease recurrence, unacceptable toxicity, or for a duration of up to one year |
|  | Monotherapy: adjuvant treatment of adults and adolescents aged 12 years and older with stage 2B or 2C *melanoma* and who have undergone complete resection (03.2023) | Until disease recurrence, unacceptable toxicity, or for a treatment duration of up to one year |
|  | Monotherapy: treatment of MSI-H or dMMR *CRC* in adults with: unresectable or metastatic *CRC* after previous fluoropyrimidine-based combination therapy; advanced or recurrent *EC*, who have disease progression on or following prior treatment with a platinum-containing therapy in any setting and who are not candidates for curative surgery or radiation; unresectable or metastatic *gastric, small intestine, or biliary cancer*, who have disease progression on or following at least one prior therapy (12.2023) | For up to 35 cycles (approximately 2 years) or until disease progression or unacceptable toxicity |
|  | Monotherapy: adjuvant treatment of adults with *NSCLC* who are at high risk of recurrence following complete resection and platinum-based chemotherapy (09.2024) | Until disease recurrence, unacceptable toxicity or for a duration of up to one year |
|  | Combination: with carboplatin and either paclitaxel or nab-paclitaxel for first-line treatment of metastatic squamous *NSCLC* in adults (08.2019) | Subject to a two-year clinical stopping rule |
|  | Combination: with pemetrexed and platinum chemotherapy for first-line treatment of metastatic non-squamous *NSCLC* in adults whose tumours have no EGFR or ALK positive mutations (09.2019) | Subject to a two-year clinical stopping rule |
|  | Combination: with platinum and fluorouracil chemotherapy for first-line treatment of metastatic or unresectable recurrent *SCCHN* in adults whose tumours express PD-L1 with a CPS ≥1 (08.2020) | Subject to a two-year clinical stopping rule |
|  | Combination: with axitinib for the first-line treatment of advanced *RCC* in adults (08.2020) | Subject to a two-year clinical stopping rule |
|  | Combination: with platinum and fluoropyrimidine based chemotherapy for first-line treatment of patients with locally advanced unresectable or metastatic *carcinoma of the oesophagus* or HER-2 negative *GOJ* adenocarcinoma in adults whose tumours express PD-L1 with a CPS≥10 (04.2022) | Subject to a two-year clinical stopping rule |
|  | Combination: with chemotherapy (paclitaxel or nab-paclitaxel) for treatment of locally recurrent unresectable or metastatic *TNBC* in adults whose tumours express PD-L1 with a CPS ≥ 10 and who have not received prior chemotherapy for metastatic disease (09.2022) | Subject to a two-year clinical stopping rule |
|  | Combination: with lenvatinib for advanced or recurrent *EC* in adults who have disease progression on or following prior treatment with a platinum-containing therapy in any setting and who are not candidates for curative surgery or radiation (09.2022) | Subject to a two-year clinical stopping rule |
|  | Combination: with chemotherapy, with or without bevacizumab, for treatment of persistent, recurrent, or metastatic *CC* in adults whose tumours express PD-L1 with a CPS ≥1 (01.2023) | Subject to a two-year clinical stopping rule |
|  | Combination: with chemotherapy as neoadjuvant treatment, and then continued as monotherapy as adjuvant treatment after surgery, for treatment of adults with locally advanced or early-stage *TNBC* at high risk of recurrence (05.2023) | Until disease progression that precludes definitive surgery or unacceptable toxicity (neo-adjuvant); until disease recurrence or unacceptable toxicity (adjuvant) |
|  | Combination: with fluoropyrimidine and platinum-containing chemotherapy for first-line treatment of locally advanced unresectable or metastatic HER2-negative *gastric or GOJ adenocarcinoma* in adults whose tumours express PD-L1 with a CPS ≥ 1 (06.2024) | Until disease progression or unacceptable toxicity |
|  | Combination: with carboplatin and paclitaxel for first-line treatment of primary advanced or recurrent *EC* in adults (06.2025) | For six cycles, followed by up to 14 cycles as monotherapy |

ALK, anaplastic lymphoma kinase; ASCT, autologous stem cell transplant; CC, cervical cancer; cHL, classical Hodgkin lymphoma; CPS, combined positive score; CRC, colorectal cancer; CRT, chemoradiation therapy; CSCC, cutaneous squamous cell carcinoma; dMMR, mismatch repair deficient; EC, endometrial cancer; EGFR, epidermal growth factor receptor; ES-SCLC, extensive-stage small cell lung cancer; GOJ, gastro-oesophageal junction; HCC, hepato-cellular carcinoma; HER-2, human epidermal growth factor 2; MIUC, muscle invasive urothelial carcinoma; mMCC, metastatic Merkel cell carcinoma; MSI-H, microsatellite instability-high; pMMR, mismatch repair proficient; NSCLC, non-small cell lung cancer; OSCC, oesophageal squamous cell carcinoma; PD-L1, programmed death ligand 1; RCC, renal cell carcinoma; SCCHN, squamous cell cancer of the head and neck; SMC, Scottish Medicine Consortium; TNBC, triple-negative breast cancer; TPS, tumour proportion score

*Table S2: Utilisation of immune checkpoint inhibitors over time, by index drug (N=10,394)*

|  | **Number of patients (%)** | | | | | | |
| --- | --- | --- | --- | --- | --- | --- | --- |
|  | **Year ICI initiated** | | | | | | |
|  | **2018** | **2019** | **2020** | **2021** | **2022** | **2023** | **2024** |
|  | 764 | 1,033 | 1,243 | 1,501 | 1,640 | 1,980 | 2,233 |
| **ICI drug** |  |  |  |  |  |  |  |
| Pembrolizumab | 410 (53.7) | 455 (44.0) | 783 (63.0) | 896 (59.7) | 928 (56.6) | 1,197 (60.5) | 1,239 (55.5) |
| Nivolumab | 272 (35.6) | 300 (29.0) | 168 (13.5) | 207 (13.8) | 201 (12.2) | 234 (11.8) | 318 (14.2) |
| Atezolizumab | 34 (4.5) | 156 (15.1) | 122 (9.8) | 185 (12.3) | 239 (14.6) | 203 (10.3) | 231 (10.3) |
| Ipilimumab + nivolumab | 37 (4.8) | 81 (7.8) | 94 (7.6) | 106 (7.1) | 150 (9.2) | 148 (7.5) | 166 (7.5) |
| Durvalumab | 3 (0.4) | 28 (2.7) | 49 (3.9) | 54 (3.6) | 43 (2.6) | 96 (4.8) | 132 (5.9) |
| Cemiplimab | - | 6 (0.6) | 17 (1.4) | 29 (1.9) | 47 (2.9) | 55 (2.8) | 60 (2.7) |
| Avelumab | 8 (1.0) | 7 (0.7) | 10 (0.8) | 23 (1.5) | 26 (1.6) | 31 (1.6) | 32 (1.4) |
| Dostarlimab | - | - | - | - | 6 (0.4) | 16 (0.8) | 30 (1.3) |
| Nivolumab + relatlimab | - | - | - | - | - | - | 24 (1.1) |
| Durvalumab + tremilimumab |  |  |  |  |  |  | 3 (0.1) |
| Ipilimumab | - | - | - | 1 (0.1) | - | - | 1 (0.0) |
| **ICI regimen type** |  |  |  |  |  |  |  |
| ICI monotherapy | 725 (94.9) | 902 (87.3) | 897 (72.2) | 988 (65.8) | 978 (59.6) | 1,179 (59.5) | 1,254 (56.2) |
| ICI + chemotherapy | 2 (0.3) | 44 (4.3) | 183 (14.7) | 285 (19.0) | 349 (21.3) | 486 (24.5) | 561 (25.1) |
| ICI combination | 37 (4.8) | 81 (7.8) | 94 (7.6) | 106 (7.1) | 150 (9.2) | 148 (7.5) | 193 (8.6) |
| ICI + TKI | - | 6 (0.6) | 66 (5.3) | 104 (6.9) | 129 (7.9) | 125 (6.3) | 172 (7.7) |
| ICI + VEGF | - | - | 3 (0.2) | 18 (1.2) | 34 (2.1) | 42 (2.1) | 52 (2.3) |
| ICI + VEGF + chemotherapy | - | - | - | - | - | - | 1 (0.0) |

* More than 1 ICI drug

ICI, Immune checkpoint inhibitor; TKI, Tyrosine kinase inhibitor; VEGF, Vascular endothelial growth factor

Table S3: Utilisation of immune checkpoint inhibitors over time, by cancer type and treatment intent (N=10,394)

|  | **Number of patients (%)** | | | | | | |
| --- | --- | --- | --- | --- | --- | --- | --- |
|  | **Year ICI initiated** | | | | | | |
|  | **2018** | **2019** | **2020** | **2021** | **2022** | **2023** | **2024** |
|  | 764 | 1,033 | 1,243 | 1,501 | 1,640 | 1,980 | 2,233 |
| **Tumour group** |  |  |  |  |  |  |  |
| Lung and chest | 433 (56.7) | 545 (52.8) | 627 (50.4) | 718 (47.8) | 730 (44.5) | 761 (38.4) | 771 (34.5) |
| Skin | 168 (22.0) | 251 (24.3) | 255 (20.5) | 259 (17.3) | 303 (18.5) | 361 (18.2) | 358 (16.1) |
| Urology | 103 (13.5) | 165 (16.0) | 203 (16.3) | 242 (16.1) | 220 (13.4) | 288 (14.5) | 318 (14.2) |
| Upper GI | 13 (1.7) | - | 15 (1.2) | 42 (2.8) | 129 (7.9) | 202 (10.2) | 270 (12.1) |
| Head and neck | 38 (5.0) | 54 (5.2) | 75 (6.0) | 130 (8.7) | 99 (6.0) | 113 (5.7) | 116 (5.2) |
| Gynaecology | - | 2 (0.2) | 18 (1.4) | 36 (2.4) | 61 (3.7) | 85 (4.3) | 128 (5.7) |
| Breast | 1 (0.1) | 4 (0.4) | 9 (0.7) | 28 (1.9) | 20 (1.2) | 92 (4.6) | 170 (7.6) |
| Lower GI | 6 (0.8) | 6 (0.6) | 34 (2.7) | 42 (2.8) | 69 (4.2) | 64 (3.2) | 81 (3.6) |
| Haematology | 2 (0.3) | 5 (0.3) | 6 (0.5) | 4 (0.3) | 6 (0.4) | 8 (0.4) | 14 (0.6) |
| endocrine | - | - | 1 (0.1) | - | 1 (0.1) | 4 (0.2) | 2 (0.1) |
| CUP | - | 1 (0.1) | - | - | 1 (0.1) | 2 (0.1) | 2 (0.1) |
| Sarcoma | - | - | - | - | - | - | 3 (0.1) |
| CNS | - | - | - | - | 1 (0.1) | - | - |
| **Treatment intent** |  |  |  |  |  |  |  |
| Curable | 26 (3.4) | 113 (10.9) | 172 (13.8) | 203 (13.5) | 212 (12.9) | 431 (21.8) | 531(23.8) |
| Non-curable | 738 (96.6) | 920 (89.1) | 1071 (86.2) | 1298 (86.5) | 1428 (87.1) | 1549 (78.2) | 1702 (76.2) |

CNS, central nervous system; CUP, cancer of unknown primary; ICI, immune checkpoint inhibitor; GI, gastrointestinal
